# Identification of single-dose, dual-echo based CBV threshold for fractional tumor burden mapping in recurrent glioblastoma

**DOI:** 10.3389/fonc.2023.1046629

**Published:** 2023-01-17

**Authors:** Aliya Anil, Ashley M. Stokes, Renee Chao, Leland S. Hu, Lea Alhilali, John P. Karis, Laura C. Bell, C. Chad Quarles

**Affiliations:** ^1^ Division of Neuroimaging Research and Barrow Neuroimaging Innovation Center, Barrow Neuroimaging Institute, Phoenix, AZ, United States; ^2^ Department of Radiology, Division of Neuroradiology, Mayo Clinic Arizona, Phoenix, AZ, United States; ^3^ Neuroradiology, Southwest Neuroimaging at Barrow Neurological Institute, Phoenix, AZ, United States; ^4^ Early Clinical Development, Genentech, San Francisco, CA, United States; ^5^ Cancer System Imaging, The University of Texas MD Anderson Cancer Center, Houston, TX, United States

**Keywords:** DSC MRI, glioma, pseudoprogression, perfusion imaging, fractional tumor burden, CBV

## Abstract

**Background:**

Relative cerebral blood volume (rCBV) obtained from dynamic susceptibility contrast (DSC) MRI is widely used to distinguish high grade glioma recurrence from post treatment radiation effects (PTRE). Application of rCBV thresholds yield maps to distinguish between regional tumor burden and PTRE, a biomarker termed the fractional tumor burden (FTB). FTB is generally measured using conventional double-dose, single-echo DSC-MRI protocols; recently, a single-dose, dual-echo DSC-MRI protocol was clinically validated by direct comparison to the conventional double-dose, single-echo protocol. As the single-dose, dual-echo acquisition enables reduction in the contrast agent dose and provides greater pulse sequence parameter flexibility, there is a compelling need to establish dual-echo DSC-MRI based FTB mapping. In this study, we determine the optimum standardized rCBV threshold for the single-dose, dual-echo protocol to generate FTB maps that best match those derived from the reference standard, double-dose, single-echo protocol.

**Methods:**

The study consisted of 23 high grade glioma patients undergoing perfusion scans to confirm suspected tumor recurrence. We sequentially acquired single dose, dual-echo and double dose, single-echo DSC-MRI data. For both protocols, we generated leakage-corrected standardized rCBV maps. Standardized rCBV (sRCBV) thresholds of 1.0 and 1.75 were used to compute single-echo FTB maps as the reference for delineating PTRE (sRCBV < 1.0), tumor with moderate angiogenesis (1.0 < sRCBV < 1.75), and tumor with high angiogenesis (sRCBV > 1.75) regions. To assess the sRCBV agreement between acquisition protocols, the concordance correlation coefficient (CCC) was computed between the mean tumor sRCBV values across the patients. A receiver operating characteristics (ROC) analysis was performed to determine the optimum dual-echo sRCBV threshold. The sensitivity, specificity, and accuracy were compared between the obtained optimized threshold (1.64) and the standard reference threshold (1.75) for the dual-echo sRCBV threshold.

**Results:**

The mean tumor sRCBV values across the patients showed a strong correlation (CCC = 0.96) between the two protocols. The ROC analysis showed maximum accuracy at thresholds of 1.0 (delineate PTRE from tumor) and 1.64 (differentiate aggressive tumors). The reference threshold (1.75) and the obtained optimized threshold (1.64) yielded similar accuracy, with slight differences in sensitivity and specificity which were not statistically significant (1.75 threshold: Sensitivity = 81.94%; Specificity: 87.23%; Accuracy: 84.58% and 1.64 threshold: Sensitivity = 84.48%; Specificity: 84.97%; Accuracy: 84.73%).

**Conclusions:**

The optimal sRCBV threshold for single-dose, dual-echo protocol was found to be 1.0 and 1.64 for distinguishing tumor recurrence from PTRE; however, minimal differences were observed when using the standard threshold (1.75) as the upper threshold, suggesting that the standard threshold could be used for both protocols. While the prior study validated the agreement of the mean sRCBV values between the protocols, this study confirmed that their voxel-wise agreement is suitable for reliable FTB mapping. Dual-echo DSC-MRI acquisitions enable robust single-dose sRCBV and FTB mapping, provide pulse sequence parameter flexibility and should improve reproducibility by mitigating variations in preload dose and incubation time.

## Introduction

Glioblastoma (GBM) is the most aggressive and common primary malignant brain tumor in humans. Treatment includes surgical resection followed by radiation treatment and chemotherapy. Within the first 3-6 months of radiation treatment, patients may exhibit MRI findings that are consistent with tumor recurrence and/or post treatment radiation effects (PTRE) (1). Thus, an important challenge in patient management is distinguishing tumor progression from PTRE (2–4). Conventional post-contrast T1-weighted images are not capable of distinguishing tumor recurrence from PTRE because treatment-induced changes and tumor recurrence similarly present as new contrast-enhancement, most often adjacent to the surgical resection cavity and within the radiotherapy treatment field. Although tumor and PTRE share similar radiological features, they represent vastly different responses to the radiation treatment response (4). More specifically, presence of PTRE shows spontaneous stabilization and thus indicates positive response to the treatment, whereas tumor recurrence indicates treatment failure. Hence, an early differentiation between PTRE and tumor progression would improve treatment planning (4–6). Advanced imaging techniques like perfusion, diffusion, and PET imaging have proven useful for delineating tumor from PTRE after therapy (7, 8).

Dynamic susceptibility contrast (DSC)-MRI based perfusion imaging has been widely validated with image-localized histopathology and is recommended for routine use in GBM patients (9–14). The derived relative cerebral blood volume (rCBV) maps provide non-invasive interrogation of the tumor vasculature that arises from aberrant angiogenic pathways. In particular, high grade and progressive tumor regions present with elevated rCBV values, whereas rCBV is markedly lower in regions of PTRE (10, 12, 15). Several studies, across multiple sites, have leveraged localized image-guided histopathology to validate that rCBV measurements reliably differentiate regional tumor recurrence from PTRE *via* correlation with histologic tumor burden (10, 16, 17). These studies have found that when DSC-MRI data is acquired and analyzed using uniform methodology, common rCBV thresholds can be employed to identify, and regionally map, PTRE and recurrence. The application of these thresholds enables computation of images called fractional tumor burden (FTB) maps (15, 18, 19). Previous studies have used rCBV threshold of 1.0 to differentiate tumor from PTRE (18). To identify more aggressive tumors (eg: predictive of outcomes), FTB maps are often computed using two rCBV thresholds (1.0, to distinguish tumor from PTRE & 1.75, to identify aggressive tumors). Thus, the derived FTB maps enable efficient visualization of 3 FTB classes: FTB_low_ (rCBV < 1.0) representive of PTRE, FTB_mid_ (1.0 < rCBV < 1.75) highlighting tumor with moderate angiogenesis and FTB_high_ (rCBV > 1.75) demarcating aggressive tumor with high angiogenesis, each represented with a unique color (20, 21). Standardization of rCBV, transforms rCBV maps to a standard intensity scale that eliminates the variability associated with user dependent ROIs used for normalization (22). Recent studies have recommended standardization to be an important step toward workflow optimization and consensus methodology (23, 24). In this study, standardized rCBV (sRCBV) values and two thresholds were used to compute FTB maps.

DSC-MRI data is most often acquired using single gradient echo acquisitions, but contrast agent leakage effects, due to the disruption of the blood brain barrier, can reduce rCBV accuracy. To mitigate these effects, a preload of contrast agent and/or post-processing leakage correction algorithms can be used (25). Recently, two alternative acquisition strategies have been proposed to mitigate the need for multiple contrast agent doses. First, a low-flip angle, single-dose protocol was shown to provide reliable rCBV maps (26). However, the fidelity and reproducibility of the low-flip angle approach requires uniform and field strength dependent pulse sequence parameters. Alternatively, replacing the single-echo with a dual-echo pulse sequence eliminates T1 leakage effects and the need for preload dosing (25, 27–29). Residual T2* leakage effects can be removed using post-processing leakage correction algorithms. The validity of single-dose, dual-echo DSC-MRI protocols, and the derived rCBV maps, were recently validated by direct comparison to the standard double-dose, single-echo protocol (30). Favorably, the dual-echo approach decouples rCBV accuracy from pulse sequence parameters, enabling greater pulse sequence parameter flexibility and improved reproducibility (30). While these studies demonstrated high accuracy for single-dose rCBV, there is a compelling need to further establish dual-echo DSC-MRI based FTB mapping to differentiate recurrent tumor and treatment effects.

In this study, we determine optimum rCBV thresholds for the single-dose, dual-echo DSC-MRI approach to generate FTB maps that distinguish tumor and PTRE and best match with the reference standard, double-dose, single-echo FTB map.

## Material and methods

### Patients

This retrospective study was approved by Dignity Health Institutional Review Board (IRB). Data acquisition was performed as part of a clinical standard of care scan, spanning October 2018 to November 2020. Inclusion criteria were presence of contrast enhancing lesions on the DSC-MRI imaging, surgical resection or biopsy of the mass, patient age >18 years, availability of perfusion datasets for both preload and main injection and high-grade glioblastoma. Exclusion criteria included different pulse sequence parameters (n = 9), poor injection (n = 1), susceptibility artifacts (n = 4), partial volume effects (n = 1), missing dynamic data points (n = 16) and low-grade gliomas (n = 14). After screening a total of 68 patients, 23 patients were included in this study. All patients received treatment within 6 weeks after surgery or biopsy. The time between diagnosis, following surgical resection or biopsy, and the date of the perfusion scan ranged from 4 - 32 months.

### MRI imaging protocol

All imaging studies were performed on a 3T MRI (Ingenia, Philips Healthcare, Best, Netherlands). The standard pre-contrast and post-contrast 3D anatomical T1-weighted images were obtained using a gradient echo sequence with the following acquisition parameters: TE/TR: 4.4/7.9 ms, acquisition matrix: 512 x 512, voxel size: 1.0 x 1.0 mm^2^, slice thickness: 1.0 mm, 170 sagittal slices, flip-angle: 8°. Two consecutive DSC-MRI perfusion datasets were acquired for all patients using two sequential full bolus doses of gadolinium-based contrast agent (gadobutrol, Gadavist) injections with spatial resolution of 1.75 x 1.75 mm^2^ (acquisition matrix: 128 x 128), slice thickness of 5 mm (20 axial slices), and pixel bandwidth of approximately 2 kHz. Bolus injections were administered after 30 seconds of baseline acquisition at a rate of 3 ml/s using a power injector. A dual-echo DSC protocol (TE1/TE2 = 7.4/33.6 ms; TR = 581.9 ms; FA = 75°) was performed for the first bolus injection for the evaluation of the single-bolus, dual-echo protocol. This injection serves as the preload for the standard double-dose, single-echo protocol. After a delay of 6 minutes, a second dose of contrast bolus was injected for the acquisition of the standard single-echo DSC protocol (TE/TR = 30/1400 ms; FA = 60°).

### Data analysis

For each patient, standardized relative cerebral blood volume (sRCBV) maps were generated using the commercially available, FDA-approved, clinical software plug-in, IB Neuro X2™ (Imaging Biometrics, Version 21.12, Elm Grove, Wisconsin). Since high-grade tumor disrupts the blood brain barrier and yields discrepancies in rCBV values, the Boxerman-Schmainda-Weisskoff (BSW) leakage correction was performed on both datasets to minimize T1 and T2* leakage effects (31). The sRCBV maps generated were co-registered to the respective T1-weighted post-contrast images using IB Delta Suite™ (Imaging Biometrics, Version 21.05). For semi-automated analysis, enhancing tumor region-of-interests (ROIs) were generated using IB RadTech™ (Imaging Biometrics, Version 21.05) based on ΔT1 images (post-contrast T1w image – pre-contrast T1w image).

### Statistical analysis

The sRCBV agreement between the two acquisition protocols on the mean tumor ROI was assessed by computing the concordance correlation coefficient (CCC) across all the patients. To identify optimal sRCBV thresholds that distinguish tumor and PTRE, a receiver operating characteristic (ROC) analysis was performed on the sRCBV values at the voxel-wise level across all patients. The optimal thresholds corresponding to the maximum value of accuracy (defined as the average of sensitivity and specificity) were identified from the ROC curve. The sensitivity, specificity, and accuracy were compared between the obtained optimum threshold and the histologically validated reference threshold (20, 21).

## Results

A total of 23 subjects that satisfied the inclusion/exclusion criteria were identified for the analysis in this study. There were 11 males and 12 females included in the study with an average age of 54.9 years (SD = 12.25; Range = 30 - 79).

**Figure 1:**
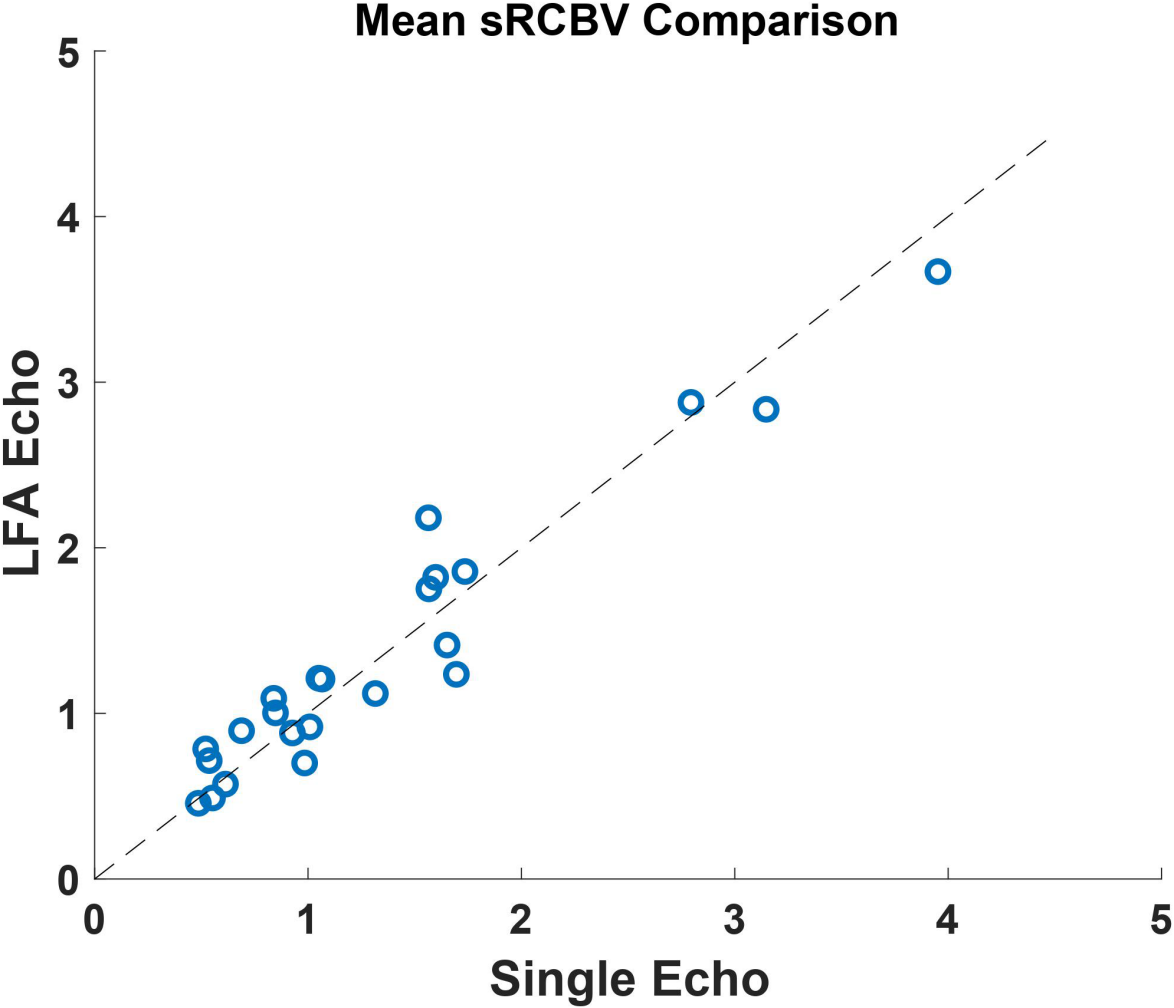
A comparison of the mean tumor ROI between the single-dose, dual-echo and the double-dose, single-echo based sRCBV values across all the patients (n=23) included in the study. This shows a strong agreement across the two protocols with a concordance correlation coefficient value of 0.96.

The sRCBV correlation on the mean tumor ROI between the single-dose, dual-echo protocol and the standard double-dose, single-echo protocol is depicted in **Figure 1**. Consistent with the prior study (26), there is strong agreement with a CCC value of 0.96 between the dual-echo and single-echo protocols.

**Figure 2:**
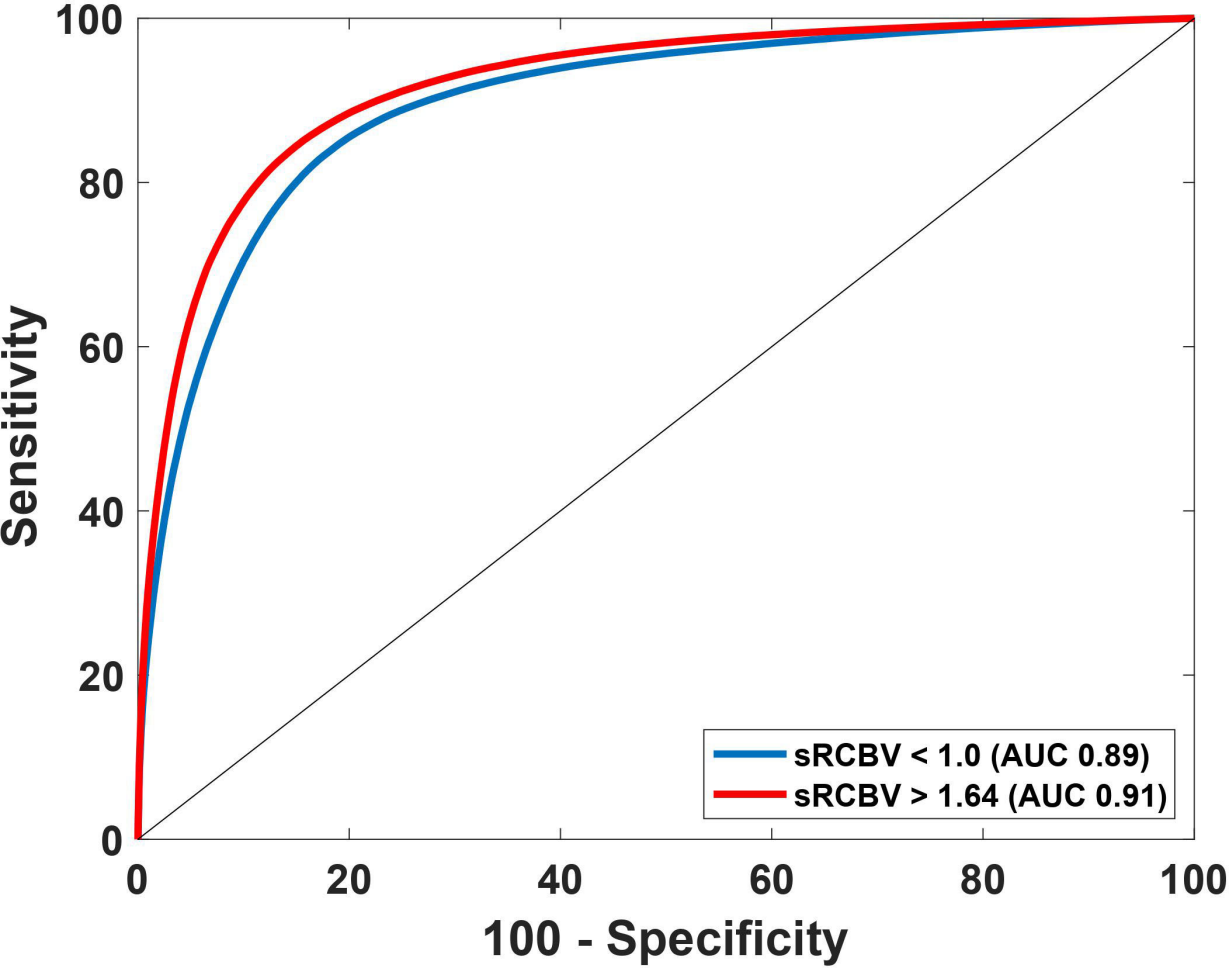
Receiver Operating Characteristics (ROC) curve using sRCBV values to identify the dual-echo thresholds. The AUROC for thresholds 1.0 and 1.64 are 0.89 and 0.91 respectively.

Using the sRCBV values, the receiver operating characteristic (ROC) curve identified the sensitivity and specificity across different thresholds as shown in **Figure 2**. The area under the ROC (AUROC) curve for sRCBV < 1.0 and sRCBV > 1.64 was 0.89 and 0.91, respectively. A lower threshold of 1.0 (delineating PTRE and tumor voxels) and an upper threshold of 1.64 (delineating tumor with high and moderate angiogenesis) provided the maximum accuracy of 82.32% and 84.73%, respectively. The sensitivity and specificity corresponding to the maximum accuracy is 87.77% and 76.88% for 1.0 threshold and 84.48% and 84.97% for 1.64 threshold, respectively.

**Figure 3:**
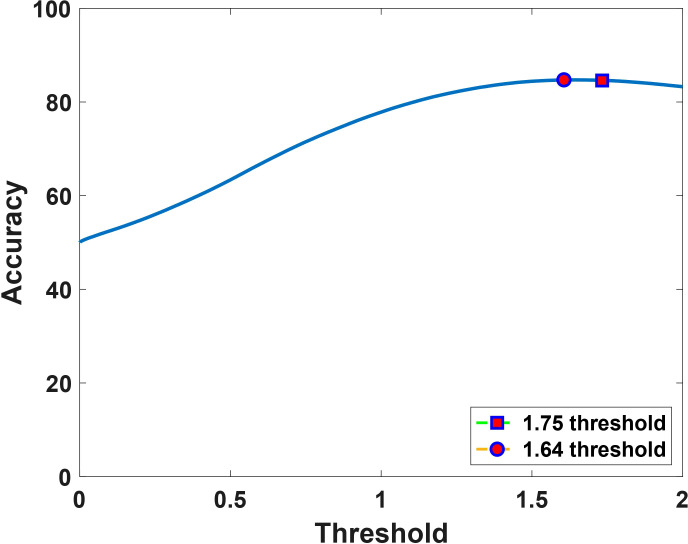
Accuracy as a function of the dual-echo derived sRCBV threshold, showing that the computed optimal upper threshold, 1.64 (marked in circle) has consistent accuracy (84%) with the reference value, 1.75 (marked in square).

According to prior studies (18), the histologically validated standard rCBV threshold to differentiate PTRE and tumor is 1.0, while 1.75 is often chosen as a marker of aggressive tumor (20, 21). By comparing the sensitivity, specificity, and accuracy between the reference threshold of 1.75 and the obtained optimized threshold of 1.64, we noted that although the sensitivity and specificity varies between the thresholds as shown in **Table 1**, the value of accuracy remains the same, which is depicted in **Figure 3**.

**Table 1:** Comparison of sensitivity, specificity and accuracy between the sRCBV threshold generated from ROC analysis (1.64) and the reference standard threshold (1.75) across 23 subjects.

ROC parameters	sRCBV threshold from ROC analysis (1.64)	Reference standard threshold (1.75)
Sensitivity	84.48%	81.94%
Specificity	84.97%	87.23%
Accuracy	84.73%	84.58%

**Figure 4:**
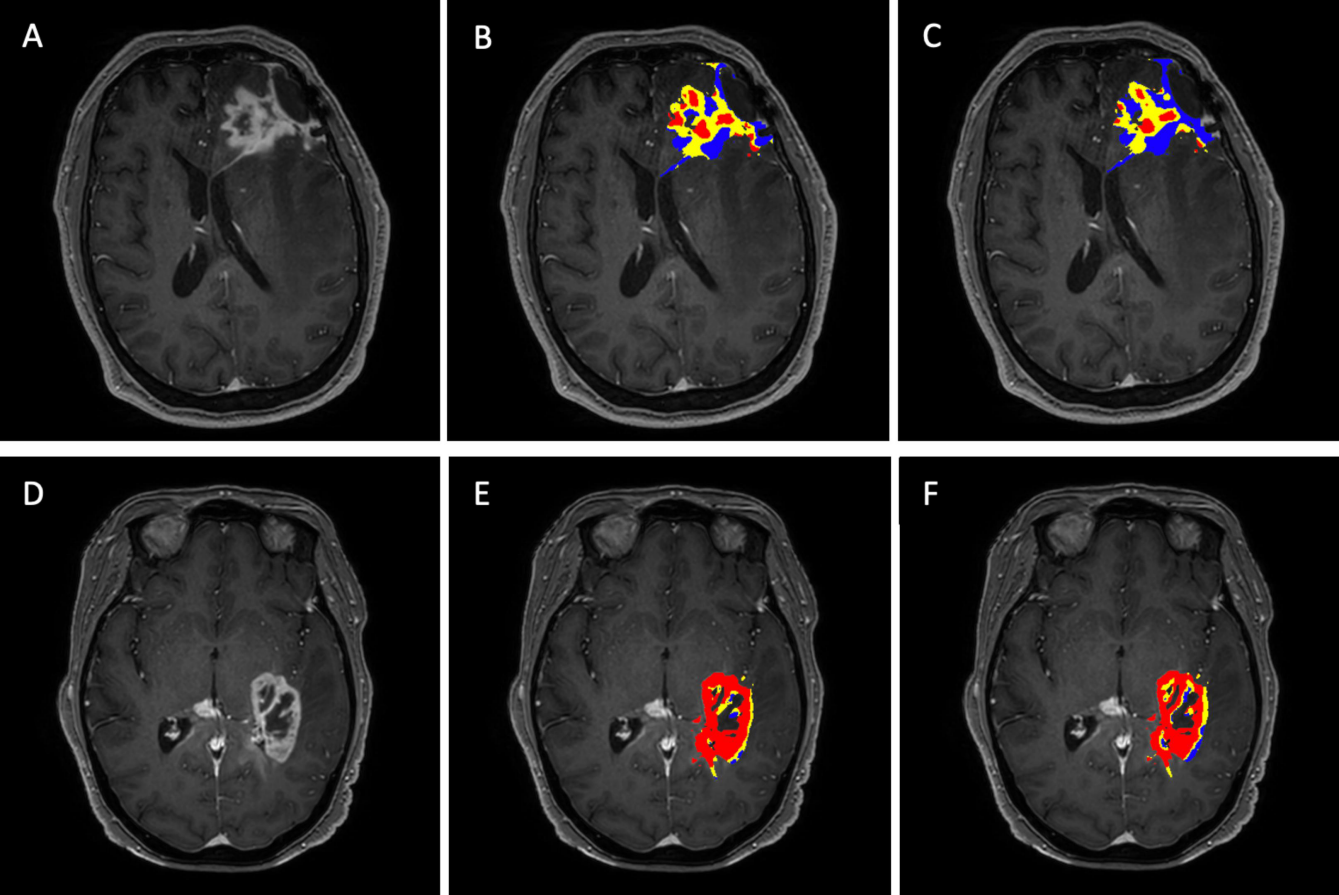
FTB maps generated for the single-dose, dual-echo and double-dose, single-echo protocol for two separate patients with the anatomical post-contrast T1 – weighted images in the far-left column **(A, D)**. Patient **(A–C)** is a 58-year-old male with grade IV glioblastoma presenting 11 months after the stereotactic radiosurgery. Patient **(D–F)** is a 65-year-old female with grade IV glioblastoma presenting 4 months after biopsy. The middle column corresponds to the FTB maps **(B, E)** generated for the single-dose, dual-echo protocol. The far-right column corresponds to the FTB maps **(C, F)** generated from the standard reference double-dose, single-echo protocol. The standard thresholds of 1 and 1.75 were used for both protocols. Blue represents areas of PTRE, yellow and red represents tumor with moderate and high angiogenesis, respectively.

**Figure 4** visually summarizes two separate cases showing the post-contrast T1-weighted images with the enhancing tumor and the corresponding FTB maps for the single-dose, dual-echo and the double-dose, single-echo protocols using the thresholds 1.0 and 1.75. The PTRE voxels are represented in blue, where the sRCBV value is less than 1.0 and is considered as FTB_low_. The sRCBV voxels between 1.0 and 1.75 are FTB_mid_ in yellow, considered as tumor with moderate angiogenesis. The FTB_high_ constitute the tumor voxels with high angiogenesis in red with sRCBV values greater than 1.75. Visually, the FTB maps across the two protocols are in strong agreement, each identifying the same regions of tumor and PTRE.

## Discussion

The use of DSC-MRI, with the double contrast agent dose and single echo pulse sequence, has been validated for distinguishing tumor recurrence from treatment related effects like pseudoprogression or radiation necrosis (25, 27, 32). The accuracy of the computed CBV maps derives from the reduction of T1 leakage effects, from the contrast agent preload, and T2* leakage effects using the BSW leakage correction algorithm (33). Similarly, dual-echo pulse sequences enable reliable CBV mapping with a single contrast agent dose through elimination of T1 leakage effects and mitigation of T2* effects using the BSW correction (30, 34). This reduces the contrast agent dose, provides significant flexibility in pulse sequence parameters, and improves reproducibility by mitigating the variation in the preload dose and incubation time (30). In a study using a patient-based and validated DSC-MRI digital reference object (DRO), multi-echo acquisitions have shown more robust results than single-echo protocols as it essentially decouples both TR and FA from rCBV accuracy. This leads to a wide range of pulse sequence parameters to yield robust rCBV maps (34). Further, as previously shown dual-echo acquisitions enable simultaneous acquisitions of DSC- and DCE-MRI data, enabling quantification of tumor permeability *via* the contrast agent transfer constant known as K^trans^ (35, 36). In this study, we aimed to further expand the clinical utility of dual-echo DSC-MRI by determination of a dual-echo derived voxel-wise CBV threshold for FTB mapping by comparison to that computed using the double-dose, single-echo standard.

To facilitate the clinical translation of the derived threshold, we employed FDA- approved clinically available software that has the capability of deriving CBV maps from single and dual-echo DSC-MRI data. In the analysis, we also leveraged standardization of rCBV maps, as this eliminates the variability associated with user-dependent ROIs for normalization (22). Prior studies have demonstrated that standardized rCBV values increase CBV reproducibility across patients and sites (22–24). Recent studies have also shown that standardization of rCBV, derived from single-echo data, achieves similar accuracy when compared with the normalized rCBV in differentiating recurrent tumor from PTRE (23).

Our study further corroborates the strong agreement between the mean sRCBV tumor values across the single-dose, dual-echo and double-dose, single-echo protocols (30). Agreement at the voxel-wise level is necessary for reliable FTB mapping. For the reference single-echo protocol, the histologically validated sRCBV threshold to distinguish tumor and PTRE is 1.0, and other studies employ an upper threshold of 1.75 as a marker of aggressive tumor (18, 20, 21, 23). ROC analysis performed on the dual-echo sRCBV identified 1.0 and 1.64 as the optimum thresholds yielding maximum accuracy. When the accuracy of the upper threshold of 1.64 was compared with the reference standard 1.75 for the dual-echo protocol, the accuracy remained effectively the same.

Therefore, with the standard threshold, the single-dose, dual-echo approach yields FTB map that strongly agree with the reference standard, single-echo FTB map, providing a compelling option to reduce contrast agent dose and improve standardization *via* dual-echo acquisitions in a clinical setting.

In conclusion, this study demonstrates that the single-dose, dual-echo protocol can be reliably used to distinguish tumor and PTRE. Validation in a larger sample size and across MRI vendors and sites would further strengthen the clinical utility of dual-echo DSC-MRI. Eventhough the well-validated double-dose, single-echo protocol generated FTB maps was used as the ground truth reference, one of the limitations of the study was the lack of histopathology correlation. An important finding of this study is the similarity between the single-dose, dual-echo FTB maps and the standard double dose, single-echo FTB maps using the standard sRCBV thresholds (1 and 1.75). This indicates that existing clinical software for FTB mapping can be reliably applied to dual-echo DSC-MRI data to diagnose and quantify recurrent high-grade tumor from treatment effects. Given that dual-echo acquisitions eliminate the need for a preload injection, decouples rCBV accuracy from pulse sequence parameters, and is more reliable across a range of brain tumor types, this study provides further motivation to continue its refinement and use in clinical studies and trials.

## Data availability statement

The raw data supporting the conclusions of this article will be made available by the authors, without undue reservation.

## Author contributions

CQ acquired the funding for the study. CQ, AS, LH and LB designed the studies. CQ, AS, LA and JK guided interpretation of results. AS, LA and JK were responsible for data acquisition. AA and RC were responsible for data analysis and AA for manuscript preparation. All authors contributed to the article and approved the submitted version.
